# Microdebrider complications in sinus surgery: Analysis of the openFDA database

**DOI:** 10.1002/wjo2.89

**Published:** 2023-01-31

**Authors:** Elias S. Saba, Jacob Hoerter, Jeremy Chang, David W. Chou, Chris Xiao, Jacob G. Eide, Rijul S. Kshirsagar, James N. Palmer, Nithin D. Adappa

**Affiliations:** ^1^ Department of Head and Neck Surgery Kaiser Permanente Oakland Medical Center Oakland California USA; ^2^ Department of Otorhinolaryngology‐Head and Neck Surgery, Perelman School of Medicine University of Pennsylvania Philadelphia Pennsylvania USA; ^3^ Department of Otolaryngology‐Head and Neck Surgery Mount Sinai Medical Center New York New York USA; ^4^ Department of Otorhinolaryngology‐Head and Neck Surgery University of Pennsylvania Philadelphia Pennsylvania USA

**Keywords:** device failure, microdebrider, openFDA, outcomes, sinus

## Abstract

**Objective:**

Functional endoscopic sinus surgery is a commonly performed otolaryngologic procedure that often uses the microdebrider device for tissue removal. Given the ubiquitous nature of the instrument, we sought to better define the patterns of device failure using the postmarket surveillance openFDA database.

**Methods:**

The openFDA database was queried for all microdebrider‐related adverse events from January 1, 2000 to November 1, 2020. Descriptive information on the nature of device failure and any associated patient injury was compiled. Reports not directly related to device failure were excluded from the analysis.

**Results:**

A total of 641 events were included in the analysis. The most common device failure was overheating (*n* = 348, 54.3%), followed by material separation (*n* = 173, 27%), and inconsistent device activation (*n* = 52, 8.1%). Of the reported events, the vast majority did not result in patient harm (*n* = 579, 90.3%). On review of the remaining cases, only 24 events (3.7%) resulted in true harm to the patient, defined as a temporary or permanent injury or >30 min of additional anesthesia time. Of these cases, the need to reschedule surgical cases (*n* = 5, 0.8%), retained foreign body (*n* = 5, 0.8%), and thermal tissue injury (*n* = 3,0.5%) were the most common. Five patients suffered an injury due to surgeon error unrelated to device malfunction (*n* = 5, 0.8%).

**Conclusions:**

Microdebrider device failures are extremely rare. When they do occur, less than 10% result in patient harm. In cases of patient harm related to microdebrider failure, preoperative testing of the device before use could prevent many of the reported malfunctions.

## Highlights

- The openFDA database was queried for adverse events related to microdebrider use over a 20‐year period.
- Analysis of the openFDA database demonstrates that microdebrider device failures are rare, and in over 90% of cases do not result in patient harm.
- The most common reasons for microdebrider device failure are overheating and device separation, and are likely related to component wear after repeated use or improper maintenance.

## INTRODUCTION

Functional endoscopic sinus surgery (FESS) is one of the most common procedures performed by otolaryngologists, and over 250,000 sinus operations are performed annually in the United States.^1^ Over the past 20 years, microdebriders have become an essential tool in the Otolaryngologist's armamentarium. The combination of suction with a rotating blade makes ablating nasal polyps and other sinonasal soft tissue extremely efficient.^2^ Today, several companies offer microdebrider instruments for surgical use.

It is imperative that surgeons understand the possible adverse events related to the use of their surgical tools. Much has been published about risks and complications related to sinus surgery, especially given the proximity of the paranasal sinuses to the orbit and brain.^3^, ^4^, ^5^, ^6^, ^7^, ^8^, ^9^ However, complications specifically related to microdebrider use have been much less commonly explored, and to our knowledge, there are no research articles characterizing patterns of microdebrider device malfunction.^10^, ^11^, ^12^, ^13^

In 2014, the US Food and Drug Administration (FDA) launched openFDA, an open‐source database that provides access to publicly available medical device reports, enforcement reports, and drug event reports (https://open.fda.gov/about/). The database has been used to explore complications related to tonsillectomy, balloon sinuplasty, nasopharyngoscopy, posterior nasal nerve cryoablation, and dermal filler injection.^14^, ^15^, ^16^, ^17^, ^18^, ^19^ Using openFDA, we sought to investigate and characterize adverse events related to microdebrider use and device malfunction in sinus surgery. Specifically, we identified manufacturers, device models, and types of malfunctions and adverse events related to microdebrider use.

## MATERIALS AND METHODS

OpenFDA is a tool that retrieves and formats data from several FDA postmarket surveillance data sources: FDA Adverse Reporting System, Manufacturer and User Facility Device Experience (MAUDE), Recall Enterprise System, and others. Medical device manufacturers, user facilities, and importers are required to submit adverse event reports to MAUDE per FDA regulation 21 CFR Part 803. Health professionals, caregivers, and patients can also voluntarily submit reports.^16^

The OpenFDA database was queried for any adverse events while using microdebrider in otolaryngologic surgery from January 1, 2000 to November 1, 2020. Device events were obtained based on the criteria listed in Table 1. The final query appeared as follows: https://api.fda.gov/device/event.json?api_key=[***redacted***]&search=device.openfda.medical_specialty_description:%22ear%22+AND+date_received:[2000‐01‐01+TO + 2020‐11‐01]+AND+(device.generic_name:%22microdebrider%22+device.brand_name:%22microdebrider%22+device.brand_name:%22Straightshot%22)&limit=1000. The following device codes were included in the original query but yielded no additional contributions: Coblation, _topaz, Polypvac, Reprocessed_Topaz_Microdebrider, _0.8 mm, and XPS_micro.

**Table 1:** OpenFDA query criteria and corresponding codes.

Query term	Desired criteria	Code
Specialty	Otolaryngology	Device.openfda.
		Medical_specialty_description:“Ear”
Time range	January 1, 2000–November 1, 2020	Date_received:[20000101+TO+20201101]
Device	Microdebrider	Device.generic_name:microdebrider+
		Device.brand_name:“Microdebrider”+
		Device.brand_name:“Straightshot”

The acquired data set was exported into Microsoft Excel (Microsoft Corporation) and manually verified for accuracy. An adverse event was defined as an event that led to patient harm, provider harm, surgical delay, device failure without harm or delay, or a near miss. The narrative section of the event report was used to categorize the event. Events that did not fit these criteria were excluded from the sample. The type of failure, aspect of the device that failed, device brand, and type of patient harm were noted. Reported events that were not categorized as adverse events were also reviewed by these criteria. No individual patient data were collected during the completion of this project. This study was deemed exempt from institutional review board review given that no human subjects were involved, and all medical device adverse event information used is in the public domain.

## RESULTS

A total of 693 adverse events were identified. These events were reviewed to ensure that they involved true adverse events involving microdebrider use in sinus surgery, and 641 events were ultimately included in our study. Of the 52 cases rejected, the most common reasons featured cases not involving microdebriders (*n* = 28), duplicate event submissions (*n* = 18), cases involving usage of a microdebrider for nonotolaryngologic purposes (*n* = 3), and cases due to surgeon error unrelated to the usage of the microdebrider (*n* = 2).

The 641 events included in our analysis featured a variety of microdebrider handpieces, blades, and burr attachments (Table 2). The most common microdebrider reported in the database involved the Medtronic M4 microdebrider (337 events). Adverse events were categorized by type of device failure (Table 3). The most common device failures included overheating (348 events), material separation or bending (173 events), and inconsistent device activation (52 events).

**Table 2:** Device involved in the adverse event.

Device type	Quantity
Handpiece	
Medtronic M4	337
Medtronic M5	88
ESSx handpiece	59
Drill 80K Visao	2
Blade	
Straight blade	91
Angled blade	42
Burr	11
Unlisted	11
Total devices	641

**Table 3:** Type of device failure.

Type of device failure	Quantity
Overheating	348
Material separation or bending	173
Inconsistent activation or speed	52
Keying/self‐activation	24
Abnormal vibration/noise	19
Component missing or exposed	9
Surgeon user error	5
Fluid leak or irrigation problem	5
Unknown/unlisted error	4
Interference with other devices	1
Residue noted after decontamination	1
Total events	641

Adverse events were then categorized based on the level of harm to the patient or healthcare provider. Of the 641 adverse events, 579 (90.3%) involved cases of device failure without patient harm. A total of 35 events (5.5%) involved device failure, resulting in a brief (<30 min) or unspecified but assumed surgical time delay. Twenty‐four events (3.7%) involved actual harm to the patient, defined as a temporary or permanent injury such as increased bleeding, undesired tissue trauma, or significant additional anesthesia exposure (>30 min). In two adverse events, the surgeon suffered a temporary injury in the form of an electric shock as the result of a device malfunction. Neither of these incidents resulted in permanent injury to the healthcare provider. In one near‐miss adverse event (0.2%), device overheating resulted in the melting of a portion of the drapes overlying the patient, with no injury to the patient.

In analyzing the 24 events involving true harm to the patient, 18 (75%) involved patient harm due to device malfunction (Table 4). Notably, five events (20.8%) featured patient harm due to surgeon error with submission report and postoperative device analysis showing no fault in the microdebrider device itself. These involved, for example, accidental injury to the anterior ethmoid artery or burn injury to the patient's nostril or lip.

**Table 4:** Type of patient harm event.

Type of patient harm	Quantity
Device failure	18
Need to reschedule case after intubation	5
Retained foreign body	5
Burn	3
Undesired tissue trauma (nonburn)	2
Bleeding	2
>30 additional minutes of anesthesia	1
Surgeon error	5
Bleeding	3
Burn	1
Undesired tissue trauma (nonburn)	1
Patient factors	1
Total patient harm events	24

## DISCUSSION

### Sequelae of microdebrider adverse events

The microdebrider is a commonly used tool in sinus surgery to remove polyps and diseased tissue. OpenFDA is a resource developed in 2013 to create drug and medical device accountability by streamlining the accessibility of publicly available data.^16^ In this study, we identify 641 adverse events associated with microdebrider use during FESS in the openFDA database. The vast majority of these events were not associated with patient or surgeon harm (90.3%). Of the remainder, the most commonly reported complication (5.5%) was a minor case delay (<30 min). In the cases where the patient was directly harmed, the need to reschedule the case after intubation (0.8%), retained foreign body (0.8%), undesired tissue trauma (0.8%), and bleeding (0.3%) were noted. To the best of our knowledge, this is the first report of microdebrider failure rates in FESS.

The advantages of microdebriders are well documented. Several studies have demonstrated the improvement in patient outcomes from microdebrider use in sinus surgery when compared to traditional instruments including decreased damage to sinonasal mucosa, faster operating times/reduced time under general anesthesia, improved surgical visibility, faster healing with reduced scarring, and reduced bleeding.^20^, ^21^, ^22^ Nonetheless, as with any surgical instrument, microdebriders have unique risks and limitations. For instance, disturbances in cardiac monitoring equipment during microdebrider device use have been reported. Patadia et al.^23^ noted several false asystolic events during microdebrider use that resulted in an aborted procedure and costly diagnostic testing, ultimately thought to be due to electrical interference from a chassis leak. Other authors have noted apparent ventricular tachycardia on intraoperative electrocardiograms, also thought to be to electrical interference.^24^, ^25^ While not directly noted in our data set, it is possible that electrocardiogram malfunction could be the cause of case rescheduling after intubation in some patients, although these details were not available in the openFDA data set.

### Etiology of microdebrider device failure

The most common reasons for failure overall were overheating and device separation. Presumably, these issues are related to component wear after repeated use or improper maintenance. It is unknown from the data how long the affected devices had been in use and if there had been prior issues with the device; unsuccessful attempts were made to obtain recommendations from device manufacturers regarding device longevity and indications for replacement. Naturally, appropriate device maintenance could prevent some of these issues, although it is difficult to make generalizations without more granular data. In the cases of patient harm, the failures occurred in a similar pattern. Retained foreign body implies some amount of device separation and thermal injury implies device overheating.

Fortunately, while the rate of device failure is low, the fact that devices generally fail due to component separation or overheating implies that safety measures can decrease adverse events. Troubleshooting techniques may depend on the manufacturer and model type of the device employed; however, some general principles related to patient safety and device care are shared by multiple microdebrider devices. Microdebrider devices should be briefly inspected and tested outside of the patient at the desired speed before in vivo use. Attention to the microdebrider for wear and corrosion, attachment of the bit to the handpiece, and test of the function of the instrument during assembly would be helpful to prevent patient injury. The use of accessories such as certain blades may cause “wobble” of the tip, suggesting that the accessory is improperly seated within the device and should be corrected before use. Commonly, the blade opening of the device may become obstructed by soft tissue intraoperatively. In this instance, the tip of the device should be placed in sterile water with a suction connected to the handpiece to remove the obstruction. Care should be taken to not submerge the hub of the device. If continued difficulties are encountered, the device should be evaluated or replaced. Overall, the microdebrider failure rate is low and there is no need for additional process controls.

### Limitations

There are a number of limitations to this study. The data are retrospective and limited to the reported information in the openFDA database. Although the manufacturers are required to report device failures, individual surgeons report voluntarily to the openFDA. Unfamiliarity with the database and lack of time to report failures act as potential confounders. Similarly, there is often a lack of details regarding complications, making it difficult to make generalizations about the data. Although it has been estimated that over 250,000 sinus surgeries are performed in the United States every year, it is unknown how many cases involve microdebriders, and thus overall rates of device failure on a per‐case basis are difficult to estimate.^1^

## CONCLUSION

Although microdebriders are very safe instruments, device failures occur and most commonly involve overheating of the instrument and component separation. We suggest examining the instrument components during assembly, which could detect issues and help prevent patient adverse events.

## AUTHOR CONTRIBUTIONS

**Elias S. Saba**: Study conception/design; data collection; data analysis; manuscript drafting. **Jacob Hoerter**: Data collection; data analysis; manuscript drafting. **Jeremy Chang**: Manuscript drafting. **David W. Chou**: Manuscript drafting. **Chris Xiao**: Revisions. **Jacob G. Eide**: Revisions. **Rijul S. Kshirsagar**: Study conception/design; manuscript drafting; revisions; final approval. **James N. Palmer**: Revisions; final approval. **Nithin D. Adappa**: Revisions; final approval.

## CONFLICT OF INTEREST STATEMENT

The authors declare no conflict of interest.

## ETHICS STATEMENT

The authors assure that this manuscript represents the authors’ original work and has not been previously published or is being considered for publication elsewhere. This reflects our research in a complete manner and credits the appropriate contributions of the listed authors. It is a study based on anonymized device‐related data obtained from a publicly available database and does not require International Review Board approval.

## Data Availability

The data that support the findings of this study are openly available in the publicly available openFDA database at https://open.fda.gov.
